# Phenolic Compounds Diminish Antibiotic Resistance of *Staphylococcus Aureus* Clinical Strains

**DOI:** 10.3390/ijerph15102321

**Published:** 2018-10-22

**Authors:** Maria Miklasińska-Majdanik, Małgorzata Kępa, Robert D. Wojtyczka, Danuta Idzik, Tomasz J. Wąsik

**Affiliations:** Department of Microbiology and Virology, School of Pharmacy with the Division of Laboratory Medicine in Sosnowiec, Medical University of Silesia in Katowice, ul. Jagiellońska 4, 41-200 Sosnowiec, Poland; maria.miklasinska@o2.pl (M.M.-M.); mkepa@sum.edu.pl (M.K.); rwojtyczka@sum.edu.pl (R.D.W.); didzik@sum.edu.pl (D.I.)

**Keywords:** polyphenols, antibacterial activity, *Staphylococcus aureus*, antibiotics

## Abstract

There is a growing body of evidence that flavonoids show antibacterial activity against both Gram-positive and Gram-negative bacteria. The mechanisms of action of phenolic compounds on bacterial cell have been partially attributed to damage to the bacterial membrane, inhibition of virulence factors such as enzymes and toxins, and suppression of bacterial biofilm formation. What is more, some natural polyphenols, aside from direct antibacterial activity, exert a synergistic effect when combined with common chemotherapeutics. Many studies have proved that in synergy with antibiotics plant flavonoids pose a promising alternative for therapeutic strategies against drug resistant bacteria. In this review most recent reports on antimicrobial action of polyphenols on *Staphylococcus aureus* strains are described, highlighting where proven, the mechanisms of action and the structure–activity relationships. Since many reports in this field are, to some extent, conflicting, a unified in vitro and in vivo susceptibility testing algorithms should be introduced to ensure the selection of effective antibacterial polyphenolic compounds with low cytotoxicity and minimal side effects.

## 1. Introduction

Discovery of antibiotics has been one of the greatest medical achievements of the twentieth century. Regrettably, their excessive, unreasonable, and inappropriate use has led to the selection and expansion of resistant bacterial strains and dramatically increased treatment failure ratio. Bacteria have developed many different mechanisms of resistance such as: (1) modification of the antibiotic binding site; (2) production of enzymes which can degrade or change the antibiotic structure; (3) mutations in genes encoding transport proteins resulting in cell wall permeability disruptions; (4) active pumping out of the antibiotics molecules [1]. In the light of the fact that the PDR (pandrug-resistant) bacterial strains resistant to all available antibiotics are being isolated all over the world the notion that the golden age of antibiotics is over and we entered the “post-antibiotic era” is fully justified.

In the European Union nosocomial infections affect approximately 3 million people each year, of which about 50,000 cases are fatal (data of European Centre for Disease Prevention and Control), and multi-drug resistant staphylococci are one of the most common cause of nosocomial infections [2]. Staphylococci are pathogens which habitually colonize the human body and significantly increases the risk of nosocomial infection, particularly for hospitalized and immunocompromized patients [2,3,4,5]. Nowadays, in all countries staphylococci are one of the major public health problems. Errors in the anti-staphylococcal treatment strategies resulted in the selection and spread of drug resistant strains. Methicillin resistant *Staphylococcus aureus* (MRSA) strains pose a serious treatment problem among hospitalized patients for their multi-drug resistance character. Moreover, staphylococcal strains resistant to glycopeptide antibiotics, which are the drugs of the last resort against MRSA strains, have already been recognized. The subsequent problem in staphylococcal infections is an increasing resistance to macrolide, lincosamide, and streptogramin B (MLS_B_) antibiotics as a consequence of their extensive use against Gram-positive bacteria [6].

Rapid emergence, selection, and spread of antibiotic-resistant bacteria command the need for the search for new treatment strategies for MDR (multi-drug resistance) infections. Thus the discovery of alternative antimicrobial agents acting through new mechanisms remains an urgent mission, but despite numerous efforts undertaken in search for new treatment strategies against multi-drug resistant infections, this goal is far from being achieved yet [7]. Because the development and implementation of a new antimicrobial drug is a difficult, time-comsuming, and very expensive process, and bacterial abilities to evolve resistance mechanisms are swift and virtually unlimited, it looks that we have approached the solid wall in finding the new classes of antibiotics and/or their chemical derivatives on which the new therapies can be based. In the ongoing battle against multi-drug resistant bacterial strains the search for, and implementation of, natural substances that may enhance the antibacterial activity of common antibiotics represents the promising alternative [8,9]. From the earliest times, many plant-derived compounds have been used in the treatment of human diseases thanks to their healing properties. The last decade resulted in numerous reports indicating that plant-isolated natural compounds in the combination with commonly used antibacterial drugs may constitute a new strategy against infections caused by the multi-drug resistant bacteria. It has been documented that plant-derived polyphenolic compounds such as flavonoids or phenolic acids demonstrate antimicrobial properties against a broad spectrum of microorganisms, sensitize multi-drug resistance strains to the bactericidal or bacteriostatic antibiotics, and are promising weapons in the natural antimicrobial arsenal [8,9,10,11,12,13,14,15,16,17,18,19]. The enhancement of the antibacterial action of antibiotics by natural compounds can be explained by different mechanisms such as: multi-target action where each compound acts on a different site in the bacterial cell; pharmacokinetic or physicochemical properties such as an increase of solubility or bioavailability of the antibiotics; or aimed for a specific bacterial resistance mechanism [20].

## 2. Polyphenols

### 2.1. Structure and Division of Polyphenols

Polyphenols exhibit antioxidant, anti-allergic, anti-inflammatory, anticancer, antihypertensive, and antibacterial properties. Due to their chemical structure polyphenols are divided into two major classes: flavonoids and non-flavonoids. The common structure of flavonoids is a carbon skeleton of diphenyl propanes and two benzene rings (ring A and B) joined by a linear three-carbon chain (Table 1). The pyran ring (ring C) is formed by an A benzene ring and by a central three-carbon chain. Flavonoids are divided into many subclasses depending on the degree of oxidation of the central pyran ring. To date, more than 8000 flavonoids have been identified. By reason of molecular structure, flavonoids can be divided into following groups: flavonols, flavones, flavanols, flavanones, anthocyanidins, and isoflavonoids. Non-flavonoids are divided into benzoic acid derivatives such as gallic or protocatechuic acid, cinnamic acid derivatives such as caffeic, ferullic, or coumaric acid and stilbenes such as resveratrol (Table 1) [21,22,23].

### 2.2. Structure-Antibacterial Activity Relationship

**Figure 1:**
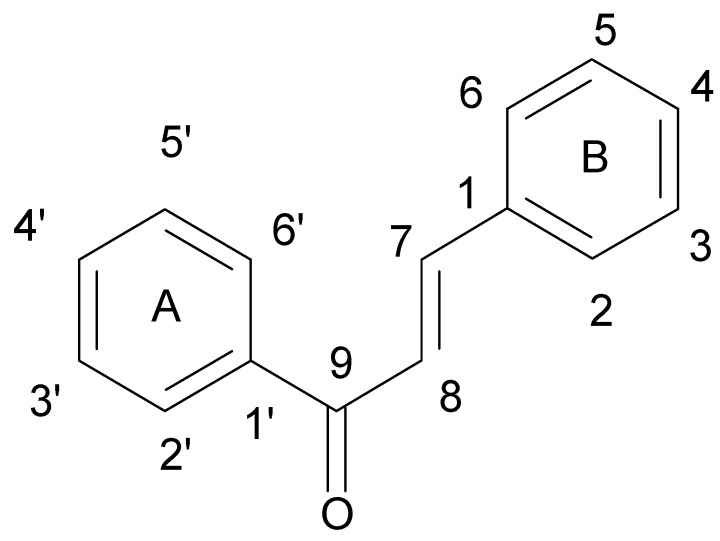
Chemical structure of chalcone.

Flavonoids comprise a group of compounds structurally diverse. Despite the fact that studies on the relationship between the structure and antimicrobial properties of flavonoids began in the early nineties of the last century, research and development works have not solved this problem [11]. Among flavonoids, the relationship between the structure and the antimicrobial activity was extensively studied in chalcones {(2*E*)-1,3-diphenylprop-2-en-1-one}. A characteristic feature of the chalcone molecule is an open heterocyclic ring, the closure of which transforms chalcone into a flavanone (Figure 1). The present findings suggest that the chalcone sceleton is an attractive template for chemical modifications which could enhance the antimicrobial potential [24].

It is known that hydroxylation of the A ring, especially in the 2′ position, augments the antibacterial properties, while the carbon substitution at 2′ position by methoxy or acetyl groups give the opposite effects. The fluoridation of the A ring in 3′ and/or 5′ position reduces the antibacterial properties too [24,25,26]. As far as the chalcone ring B is concerned, it is known that the attachment of lipophilic substituents, especially at the 4′ and 5′ position, enhances the antibacterial potential [27,28]. The presence of phenolic hydroxyl groups with high protein binding affinity may inhibit microbial enzymes and simultaneously increase affinity to cytoplasmic membranes, thus enhancing the antibacterial activity. It has been shown that substitution of single hydroxyl group and/or certain degree of the lipophilicity to the compound is sufficient enough to increase its antibacterial properties [24].

Moreover, it has been proven that hydroxyl substitution at the 2′ and 4′ or 2′ and 6′ positions of the B-ring and at the 5′ and 7′ positions of the A-ring significantly increases antibacterial activity of flavanones even against the MRSA strains. The enhancement of antimicrobial potential can be achieved also by substituting the 6′ or 8′ position with a long aliphatic chain [11,29,30].

The structure–activity relationship was further explored by Alcaraz et al. [31] who demonstrated that the presence of a hydroxyl group in the carbon 5′ of a ring A of flavanones and flavones increases the effectiveness of these compounds against MRSA strains, while the methoxy groups exert an opposite effect and reduce the antimicrobial activity. This observation has been confirmed by the Smejkal et al. [32] and Alcaraz et al. [31] who showed that chalcones exert greater activity towards MRSA strains than flavanones and flavones, and the substitution of the hydroxyl group at the 5′ position of these compounds further enhances the antibacterial effect.

The substitution of the hydroxyl group at the 3′ carbon of the C-ring of flavonoids increases the antibacterial activity. [11,32]. In addition, the O-acyl or O-alkyl chains in the above position also augment antibacterial activity, especially of flavonols and flavanols [11,33]. There are also reports that the substitution of sulfur or nitrogen in 4′ position of the C ring may also ameliorate antimicrobial activity [11,34].

In summary, an increase in the hydrophobicity of the flavonoids by long aliphatic chains substitution facilitates interactions with the bacterial cytoplasmic membrane, thus increases antibacterial activity of these compounds. What is more, the presence of phenolic hydroxyl groups, which have high affinity for proteins, and microbial enzyme-inhibition may enhance antibacterial effects of flavonoids through another mechanism. As aforementioned [29,30,31,32,33,34], hydroxylation of flavonoids improves antibacterial activity even against MRSA strains.

Other studies also demonstrated that synthetic modifications of natural flavonoids increase their antibacterial activity. For example, the addition of the N-heterocyclic ring in 7′ position of the ring A of chrysin yields 16–32-fold increase in antimicrobial activity compared to the chrysin itself [11,35], because its greater lipophilicity. Liu et al. [36] showed that quercetin and chrysin possess a stronger antimicrobial activity against *S. aureus* ATCC 6538 strain (minimal inhibitory concentration (MIC) = 6.25 μg/mL) than luteolin (50 μg/mL) and other 140 glycoside derivatives (100–400 μg/mL), because of their relatively low polarity [36].

## 3. Antistaphylococcal Phenolic Compounds

Polyphenols exhibit antimicrobial activity against broad spectrum of bacteria. Among the polyphenols, flavanols, flavonols and phenolic acids possess the highest antibacterial activity thanks to the ability to (1) inhibit bacterial virulence factors such as enzymes and toxins, (2) interact with cytoplasmic membrane (3) suppress biofilm formation and (4) exert a synergistic effect with antibiotics [21,22,23].

It is not unambiguous whether flavonoids have bactericidal or bacteriostatic effects. This problem has been explored by several groups of researchers. The time-kill test or MBC (minimal bactericidal concentration) assays demonstrated that compounds such as epigallocatechin gallate [37], galangin [38] and 3-*O*-octanoyl-(+)-catechin [39] caused eradication of bacterial cells of MRSA-YK, *S. aureus* NCTC 6571 and EMRSA-16 strains. On the other hand, 3-*O*-octanoyl-(−)-epicatechin also produces pseudomulticellular aggregates formed in both methicillin resistant and sensitive, *S. aureus* strains [39]. The same effect has been observed in the presence of epicatechin gallate, however, it is not clear if the true or pseudomulticellular aggregates were created in the flavonoids’ presence [11,37,40]. The formed aggregates are believed to be a single colony forming units (CFU), hence the false impression of reducing the number of CFUs [11]. This type of action strongly suggests that flavonoids do not exert bactericidal action, but the formation of aggregates is responsible for reduced number of CFUs [37,38,39,40].

In this review we focused on the polyphenolic compounds with proven, significant antibacterial activity against staphylococcal strains alone and in combination with antibiotics. A survey of recently published antistaphylococcal proprieties of flavonols, flavanols and phenolic acids is given in Table 2.

### 3.1. Flavonols

**Figure 2:**
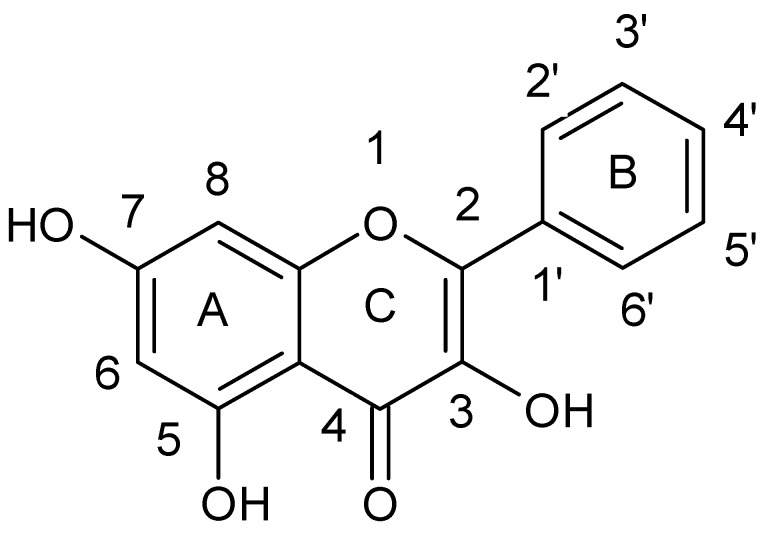
Chemical structure of galangin.

The flavonol galangin (3,5,7-trihydroxy-2-phenylchromen-4-one) is a component of propolis and *Helichrysum aureonitens* [38] (Figure 2).

Cushnie et al. [38] used electron microscopy to investigate if discussed above reduced CFUs number in the galangin presence resulted from the direct bactericidal action of this compound or from formation of cell aggregates [38]. The number of *S. aureus* NCTC 6571 colonies was diminished by approximately 15,000-fold after incubation with galangin. Under electron microscopy significant increase in the number of large clusters of bacterial cells in populations incubated with the galangin were observed while the control samples and samples untreated and treated with sodium carbonate were visible in the microscope as isolated cells, pairs or small bacterial aggregates. Because of the galangin ability to clump bacterial cells the authors suggested that this flavonoid acts directly on the cytoplasmic membrane [38]. The authors pointed out that the CFU reduction observed with the use of MBC and time-kill assays can be misinterpreted as a bactericidal effect. The aggregation of bacterial cells reduces the population’s surface area, and in consequence lowers the oxygen availability and nutrients uptake, what could be misinterpreted as metabolic enzymes or nucleic acid synthesis inhibition. It is quite possible that some studies have confused this effect with a direct antibacterial action. The future works should focus on the assessment of cell viability and divisibility in the aggregates formed by flavonoids [11,15,38].

Cushnie et al. investigated the antibacterial activity of galangin on staphylococcal cytoplasmic membrane by measuring the loss of potassium by *S. aureus* cells incubated with this flavonoid. The authors noted the 21% increase in loss of potassium after incubation with galangin, next the experiment was repeated in the presence of two antibiotics: novobiocin and penicillin G. As expected, since novobiocin is an antibiotic which inhibits DNA replication, while penicillin G is a bactericidal agent which targets the cell wall, no increase in potassium loss in the presence of novobiocin was observed, while incubation with penicillin G increased K^+^ loss by 6%. The addition of the antibiotic, which disrupts the cell wall, intensifies this effect [15,41]. The result clearly points out that galangin action is associated with ion transport across the staphylococcal cell membrane but whether the observed effect is due to direct damage of the cytoplasmic membrane or indirect damage through the binding to the cell wall that leads to lysis remains to be determined.

As above studies shown, the mechanism of action of galangin depends rather on interaction with the cytoplasmic membrane than on direct antibacterial activity. The future studies should focus on cell morphology and viability in the aggregates formed by galangin and on its interaction with antibiotics.

**Figure 3:**
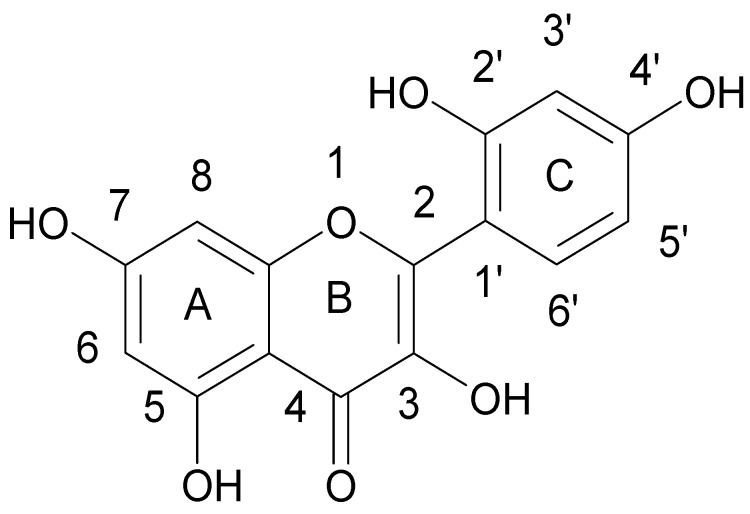
Chemical structure of morin.

Numerous studies showed the effect of flavonols on the staphylococcal virulence factors activity. Kang et al. [42] demonstrated that morin {2-(2,4-dihydroxyphenyl)-3,5,7-trihydroxychromen-4-one} (Figure 3) can inhibit sortase—the enzyme present in the cytoplasmic membrane of Gram-positive bacteria which is responsible for anchoring protein virulence factors to the cell wall peptidoglycan. Interestingly, in the presence of morin *S. aureus* shows reduced affinity to fibrinogen, which plays a key role in infections caused by staphylococci, especially in the hospital environment where binding to fibrinogen starts the biofilm formation on biomedical materials [11,42].

Except the direct antibacterial action described above, some flavonols exert a synergistic effect with antibiotics and are able to sensitize the bacteria to antibiotics to which they had previously been resistant.

**Figure 4:**
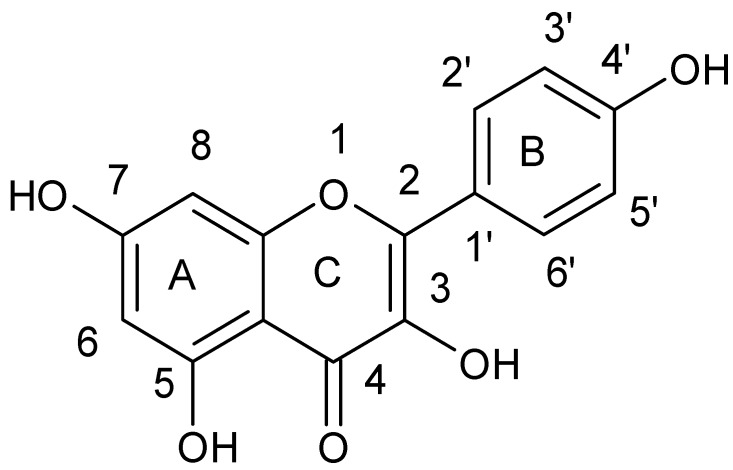
Chemical structure of kaempferol.

**Figure 5:**
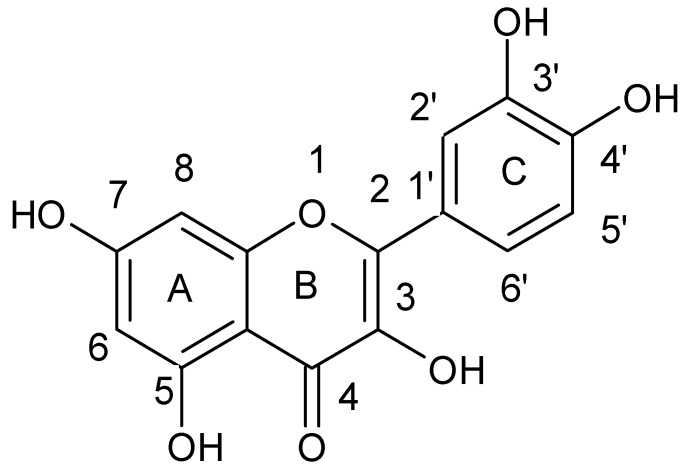
Chemical structure of quercetin.

Lin et al. [43] tested the combined effect of kaempferol {3,5,7-trihydroxy-2-(4-hydroxyphenyl)-4*H*-chromen-4-one} (Figure 4) and quercetin {2-(3,4-dihydroxyphenyl)-3,5,7-trihydroxy-4*H* chromen-4-one} (Figure 5) with rifampicin against rifampicin-resistance MRSA strains. Kaempferol and quercetin alone were able to lightly inhibit β-lactamase, but while combined with rifampicin observed inhibition increased by 57.8 and 75.8%, respectively. Moreover, the authors proved that sub-inhibitory concentrations of kaempferol and quercetin enhanced bactericidal activity of ciprofloxacin. Ciprofloxacin as fluoroquinolone causes the death of a bacterial cell by binding to *S. aureus* topoisomerase IV which leads to the inhibition of DNA synthesis. The authors suggested that the observed synergistic effect is associated with the ability of kaempferol and quercetin to inhibit the catalytic activity of different bacterial topoisomerases [44,45]. Liu et al. also showed synergistic effect of kaempferol glycosides isolated from *Laurus nobilis L*. with fluoroquinolone antibiotics against MRSA strains [46]. In their study the MIC values of ciprofloxacin against MRSA strains ranged from 0.5–64 µg/mL, while after kaempferol glycosides addition the observed MIC values were smaller and ranged from 0.13 to 16 µg/mL. The examined kaempferol glycosides potentiated the activity of ciprofloxacine 4- to 8-fold against all MRSA strains.

### 3.2. Flavanols

**Figure 6:**
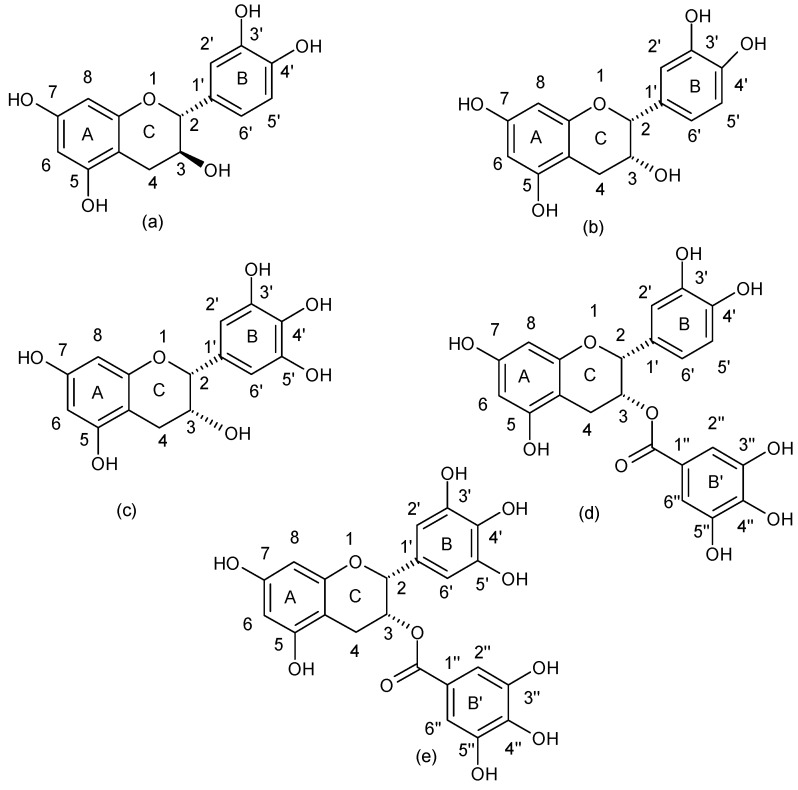
Chemical structure of (**a**) flavanols: catechin, (**b**) epicatechin, (**c**) epigallocatechin, (**d**) epicatechin gallate and (**e**) epigallocatechin gallate.

Flavanols are a well known group of flavonoids which can be found in the tea leaves. This group includes: catechin, epicatechin, epigallocatechin, epicatechin gallate, and epigallocatechin gallate (Figure 6).

The antibacterial properties of tea have been known for over a century and its bacteriostatic and bactericidal activity have been well documented. It is believed that activity of flavonols is related to their ability to bind to the lipid bilayer of the bacterial plasma membrane [47,60,61,62,63,64,65,66]. The direct antibacterial activity of alkyl gallates [67] as well as indirect action, by inhibition of the virulence factors biosynthesis of *S. aureus* such as coagulase or *α*-toxin, and reduction of mucus production and biofilm formation has been documented [37]. In addition, flavanols stimulate formation of aggregates and clumping of staphylococcal cell wall [37,68,69]. It has been also shown that the (−)-epicatechin gallate and (−)-epigallocatechin gallate can sensitize MRSA strains to *β*-lactam antibiotics [13,47,70,71,72], and that epicatechin gallate and epigallocatechin gallate act as a *norA* gene suppressors [73] and diminish *β*-lactam MICs to the antibiotic breakpoint, thus enhance the antibacterial effects of those *β*-lactams [4,13,71,73].

(−)-Epigallocatechin gallate (EGCG) ability to augment the antistaphyloccocal activity of antibiotics has been widely tested. Many studies indicated that EGCG shows synergistic effect with many classes of antibiotics, including *β*-lactams, against MRSA strains [27,48,49,50,51,52,74,75,76]. Cho et al. showed that the green tea extract rich in polyphenols such as such as epicatechin (EC), epigallocatechin (EGC), epicatechin gallate (ECG), EGCG, and gallocatechin gallate, revealed antimicrobial action against every MRSA (13) and MSSA (17) clinical strains tested. The MICs values for oxacillin in the presence of sub-inhibitory concentration of this extract were reduced from 8 to 12 times for all MRSA strains. The 2D polyacrylamide gel electrophoresis identified 17 extracellular MRSA proteins: 14 down- and 3 up-regulated after incubation with analyzed extract. DnaK protein, similar to autolysin precursor, GroEL protein, surface protein, capsular polysaccharide synthesis enzyme Cap5G, enolase, fructose-bisphosphate aldolase homologue, translation elongationfactor, leukocidin subunit precursor, *α*-hemolysin prekursor, *β*-hemolysin, glycerophosphoryl diester-phosphodiesterase, secretory antigen SsaA homology, exotoxin 15 were down-regulated while peptidoglycan hydrolase, serine protease, immunodominant antigen A were up-regulated which suggests that examined compounds can enhance the bactericidal activity of oxacillin against MRSA by changing the MRSAs protein expression [51].

Hu et al. conducted extensive research on the combination of EGCG with antibiotics. In the first paper they tested the antimicrobial activity of the combination of ECGC in different concentrations with an ampicillin-sulbactame mixture (2:1) against 28 *S. aureus* clinical strains. The results of this work proved that the activity of antibiotics against *β*-lactamase-producing MRSA strains was augmented 4–8 and 8–32 times when combined with 6.25 or 25 μg/mL of EGCG respectively, which was the susceptibility breakpoint. The time-kill curves also showed potentiating activities of this combination against both β-lactamase-producing and non-producing *S. aureus* strains [52]. Zhao et al. conducted studies on the above strains using EGCG in combination with penicillin and oxacillin and reported that MICs values of penicillin against 25 MRSA clinical strains were reduced 2–8, 2–16, and 8–32 times after addition of 6.25, 12.5 and 25 μg/mL of EGCG, respectively, while MICs values for oxacillin decreased 4–16, 4–32, and 8–64-fold, respectively. The authors concluded that the observed antibacterial effect of the EGCG-*β*-lactam antibiotics can be attributed to concerted action where damage to the bacterial cell wall was augmented by the EGCG direct binding to peptidoglycan [47]. Zhao et al. also showed a synergistic effect of EGCG with penicillin against each of 21 penicillinase producing *S. aureus* examined strains, causing direct damage to the cell wall and the inhibition of penicillinase activity [48]. These authors also demonstrated that EGCG (in concentration 1.56–25 μg/mL) increased antibacterial activity of carbapenems (imipenem, panipenem, and meropenem) against all 24 examined clinical isolates of MRSA [50].

Sudano Roccaro et al. raported that EGCG was also able to reduce tetracyclin resistance of two clinical *S. aureus* strains [53]. The MIC values for tetracycline-EGCG combination ranged from 0.5 to 64 µg/mL, and the effect was concentration dependent, while the MIC value of tetracycline alone was 1 µg/mL for *S. aureus* tetracycline susceptible strain and 128 µg/mL for *S. aureus* tetracycline resistant strain. Another study tested the antibacterial action of EGCG in combination with oxytetracycline against eight standard and clinical multidrug resistant *S. aureus* strains. The authors pointed out that EGCG was also able to increase in 8–10 fold the susceptibility of eight standard and clinical multidrug resistant *S. aureus* strains to oxytetracycline [49]. A strong synergistic antibacterial effect between EGCG and the flavonoid quercetin was detected in MRSA strains, probably due to the co-permeabilization of the bacterial membrane [49]. Moreover, Betts et al. in their work investigated the synergistic effect of the EGCG with another flavonoid—quercetin—against MRSA strains and showed strong antibacterial effect which increased significantly after quercetin addition. The authors suggested that the observed synergy was probably related to co-permeabilization of the bacterial membrane which allowed the examined compounds inside the cell [77].

In summary, the (−)-epigallocatechin gallate exert synergistic effects with a wide range of antibiotics with different mechanisms of action. Moreover, it acts on the staphylococcal cell wall directly and/or indirectly by influencing expression of staphylococcal virulence factors such as penicillinase and thus enhancing the antibacterial effect of antibiotics.

The majority of studies on flavonoids focused on antibacterial activity of epicatechin and epicatechin gallates [48,49,50,51,52,75,76,77,78] while the reports on antibacterial action of catechin are scarce.

Some studies have demonstrated that catechins could also reverse oxacillin resistance in *S. aureus* [54,61,69,72]. Stapleton et al. tested the antibacterial activity of catechins against *S. aureus* strains and stated that MRSA strains were insensitive to (+)-catechin with MICs > 256 mg/L [13]. Nevertheless, the incorporation of the acyl chains into (+)-catechin allowed the MICs reduction to 16–256 mg/L, probably due to an increase in binding affinity to the cytoplasmic membrane [40]. These authors also demonstrated that ECG was able to sensitize MRSA strains to *β*-lactam antibiotics [37], by causing changes in the structure of teichoic acids that result in the accumulation of autolysins in the cell wall. However, the modulation of *β*-lactam resistance by ECG is not associated with a decrease in PBP proteins expression, but rather by some changes in the PBP 1 and 3 proteins, leading to a 5–10% decrease in peptidoglycan cross-linking, which seems to be not sufficient to reduce the *β*-lactam resistance [37].

Unfortunately, natural catechin gallates such as ECG are not suitable for in vivo therapeutic use as they are poorly absorbed from the intestine and are susceptible to hydrolysis by bacterial enzymes. The synthesis of modified ECG derivatives with the hydrolytically susceptible ester bond been substituted by inherently more stable amide linkage can solve this problem [54]. 

Park et al. tested the antimicrobial properties of 3-*O*-alkyl analogues of (+)-catechin against Gram-positive bacteria and showed that alkylation enhanced the activity of a parent compound and antimicrobial potential increased with the number of carbons in the alkyl chain. After alkylation ECG has gained significantly greater activity against Gram-positive bacteria and a 3-*O*-decyl-(+)-catechin derivative turned out to be much more active than the parent compound [11,55]. Moreover, Shibata et al. proved that alkylated gallates enhance the antibacterial activity of *β*-lactam antibiotics against MRSA strains when combined with four *β*-lactam and nine non-*β*-lactam antibiotics. The optimal length of the alkyl chain was of C5 and C6 length [78]. Shibata et al. tested also combinations of a short (propyl) and long (octyl) chain gallates with oxacillin and FICIs ≤ 0.031 were obtained with 25 μg/mL propyl gallate and 12.5 μg/mL octyl gallate [79].

Qin et al. tested the combined effect of catechin and epicatechin gallate with *β*-lactam antibiotics against standard and clinical MRSA strains [4]. Catechin alone did not enhance the susceptibility of tested strains to *β*-lactam antibiotics with MICs > 1024 mg/L, but in combination with epicatechin gallate increased the susceptibility of MRSA strains to *β*-lactams. Interestingly, this effect was not associated with epicatechin gallate, but with catechin concentration. In addition, catechin demonstrated higher efficiency than *cis* forms of non-galloylated catechins such as (−)-epicatechin or (−)-epigallocatechin in sensitizing MRSA strains to β-lactam antibiotics. Qin et al. [4] proved that the combination of catechin with epicatechin gallate increased the antibacterial activity of ampicillin, ampicillin/sulbactam, cefazolin, cefepime, and imipenem/cilastatin, thus antibiotics which are usually ineffective against MRSA infections. However, no synergy was observed between these flavonoids combined and non-β-lactam antibiotics. The increase in the antibacterial action of *β*-lactams generated by the supplementation with catechin-epicatechin may be due to the accumulation of antibiotics and the expression inhibition of the efflux pump gene [4]. In our previous paper we tested the antibacterial potential of catechin hydrate alone and in combination with clindamycin, erythromycin, cefoxitin, and vancomycin against 23 clinical and 3 standard strains of *S. aureus* [18] and observed a substantial MICs reduction for all antibiotics after catechin hydrate supplementation. However, it should be marked that some strains were found to be resistant to catechin hydrate-antibiotic combinations. No MIC changes after catechin hydrate addition were observed in 3 *S. aureus* strains. The level of resistance to erythromycin, clindamycin and cefoxitin was not affected by the supplementation with catechin hydrate for 7 *S. aureus* strains. Furthermore, for some strains and some antibiotics after catechin hydrate addition antagonistic interactions were noted. The most noticeable synergy was noted for catechin hydrate in combination with erythromycin and clindamycin. The enhancement of antibacterial activity of vancomycin and cefoxitin after catechin hydrate addition was also observed, but it did not prove to be statistically significant. The profile of resistance to methicillin did not influenced MICs changes. We noted that catechin hydrate is not effective against strains with constitutive phenotype of MLS_B_ resistance what suggests that observed effect is not related with *erm* genes pathways which participate in above resistance. 

Summarizing, flavonoids are a promising class of natural compounds with antibacterial activity against multi drug-resistant *S. aureus* isolates. It is important to note that the antimicrobial potential of flavonoids can be augment by chemical substituents, as discussed works have showed. Moreover, the synergism between flavonoids and antibiotics suggests the potential application of such mixtures as a novel tool for fighting multi-drug resistant infections.

### 3.3. Non-Flavonoids

**Figure 7:**
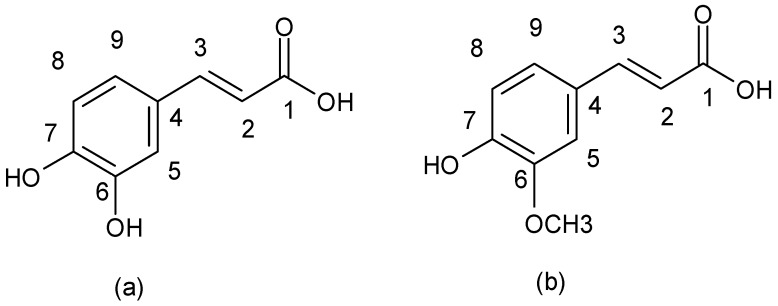
Chemical structure of (**a**) caffeic acid and (**b**) ferulic acid.

Non-flavonoid compounds are divided in phenolic acids, stilbenes, coumarins, and tannins. Phenolic acids are plant metabolites which are mainly present in chokeberry, blueberry, dark plum, cherry, coffee, and green and black teas. Many reports have proven the antibacterial properties of phenolic acids, mainly caffeic acid {3-(3,4-dihydroxyphenyl)-2-propenoic acid} and ferulic acids {3-(4-hydroxy-3-methoxyphenyl)-2-propenoic acid} (Figure 7).

Recently, Zhao et al., tested the antibacterial effect of polyphenols extracted from sugarcane where the gallic acid constituted for predominant component, followed by ferulic, coumaric acid, and chlorogenic acids. The examined mixture exerted antibacterial activity against *S. aureus* strains with MIC value at 0.625 mg/mL. The authors treated *S. aureus* cell suspensions with sugarcane bagasse extract to examine conductivity changes and thus evaluate if there is a relationship between antimicrobial potential of the extract and membrane permeability of the tested bacteria. They observed higher conductivity for strains exposed to the extract than for control strains, which suggested that the extract affects bacterial membrane integrity, causing cellular electrolyte leakage. Moreover, the authors proved that phenolic acids also change the bacterial cell morphology after incubation with a sub-inhibitory concentration of non-flavonoid polyphenols. With the use of scanning electron microscopy and transmission electron microscopy, the authors evaluated S. aureus morphology after treatment with sugarcane bagasse extract. The extract treated *S. aureus* cells showed irregular wrinkles on their surface, with fragmentation, adhesion, and aggregation of damaged cells or cellular debris. All the changes indicated that the examined extract caused extensive damage to the external structure of S. aureus cells leading to leakage of cytoplasmic components [56]. The results obtained by scanning electron microscopy and transmission electron microscopy proved that the sugarcane bagasse extract may change the morphology of staphylococcal cells and internal structure. The antibacterial activity of caffeic and ferulic acid were explored also by Borges et al. The authors showed that both acids have antibacterial activity against *S. aureus* CECT 976 strain with MIC value 1750 μg/mL for gallic acid and 1250 μg/mL for caffeic acid. MBC ranged 2500–5500 μg/mL for both compounds. Similarly to Zhao et al. they also assigned antibacterial action of gallic and caffeic acids to the damage caused to the bacterial cell wall with consecutive leakage of cellular materials [14].

An important aspect of the antibacterial properties of phenolic acids is their interaction with antibiotics. In our previous studies, the antibacterial potential of protocatechuic acid ethyl ester (ethyl 3,4-dihydroxybenzoate, EDHB) and caffeic acid (CA) alone and in antibiotic-phytochemical combination against *S. aureus* reference and clinical strains isolated from infected wounds was examined. It was demonstrated that EDHB possesses an antimicrobial activity against clinical *S. aureus* strains. The MICs of EDHB against *S. aureus* strains ranged from 64 to 1024 μg/mL. Obtained data proved significant synergistic effects between EDHB and clindamycin. Interestingly, for EDHB and cefoxitin the antagonistic trend was observed. This effect can be explained by competitive interaction, since it is very possible that cefoxitin and EDHB have the same binding site in bacterial cells [5].

Our previous paper concerning caffeic acid, showed diverse activity on *S. aureus* isolates with the minimal inhibitory concentration (MIC) varied from 256 µg/mL to 1024 µg/mL. The supplementation of Mueller-Hinton agar with ¼ MIC of caffeic acid resulted in augmented antimicrobial activity of erythromycin, clindamycin, cefoxitin and to the lesser extent of vancomycin. The observed antibacterial properties of caffeic acid seemed to be rather strain than antibiotic dependent. Data demonstrated that caffeic acid alone exerts antimicrobial activity against MSSA and MRSA strains and could potentiate antibacterial effect in combination with antibiotics. Previous reports on the antibacterial potential of caffeic acid against *S. aureus* reference strains in some cases yielded ambiguous results with the different MIC values received for the same strains [8,16,57,58,59,80,81], probably due to differences in the experiment methodology.

Luis et al. studied caffeic acid’s mechanism of action and suggested that it could be associated with polyphenol-membrane interaction. The authors noted that enhancement of the permeability and depolarization of the cell membrane and inhibition of respiratory activity in *S. aureus* ATCC 25923 strain were associated with the presence of caffeic acid. The authors proposed that caffeic acid’s mechanism of action is associated with the cell membrane damage and changes in the aerobic metabolism of *S. aureus* cells [16]. Moreover, caffeic acid as a phenolic acid possesses strong nucleophilic properties, which allows it to donate an electron pair to electrophilc functional groups of plasma membrane proteins and/or lipids, probably leading to the membrane dysfunction [82,83]. The fact that caffeic acid inhibits *α*-hemolysin secretion in *S. aureus*, the process which is membrane dependent, additionally confirms the above observations [16].

In fact, caffeic acid showed a stronger antimicrobial activity than gallic acid, vanillic acid, and protocatechuic acid [81]. Stojković et al. evaluated the potential use of caffeic acid as a food preservative against *S. aureus* contamination. The authors demonstrated that caffeic acid had an antimicrobial activity stronger than p-coumaric acid and rutin [84]. Based on our previous papers we can compare antimicrobial and combined activity of caffeic acid to other natural compounds: EDHB and catechin hydrate [5,18,57]. Results obtained by authors of the present review showed that caffeic acid possesses stronger antistaphylococcal activity than EDHB and catechin hydrate, as well as a greater synergistic effect with antibiotics than other compounds [5,18,57]. The high antibacterial activity of caffeic acid seemed to result from the presence of a propenoic side chain that reduces its polarity in comparison with the hydroxybenzoic structure of protocatechuic acid. The studies with catechins have also demonstrated that antimicrobial potential of these compounds are enhanced by the number of carbons in the alkyl chain. Summarizing, caffeic acid has the strongest antibacterial activity because of the easier transfer across the cell membrane and higher affinity to the lipid bilayer [16,82,83].

## 4. Conclusions

The present review proves that polyphenols constitute a promising source of effective, safe, and cheap antibacterial compounds. Despite the fact that most of the studies cited in this review focused on *in vitro* assays only, the antimicrobial potential of natural compounds opens a wide range of possibilities for new antibacterial therapies. Although polyphenols with MICs higher than antibiotics cannot be used in antimicrobial monotherapy as a result of their insufficient therapeutic effect, the implementation of a combined therapy with antibiotics can improve their pharmacokinetic and pharmacodynamic properties. Moreover, the use of polyphenols may also allow to reduce drug dosage and thus diminish the side effects of antibiotics. Further studies should focused on in vivo tests and clinical trials to define the usefulness of these antibacterial agents in the clinical area. 

## Figures and Tables

**Table 1:** Main classes of plant-derived polyphenols.

**Flavonoids**	
Flavonols	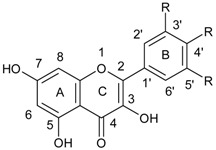
Flavones	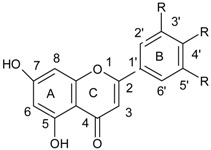
Flavanols	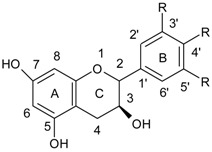
Flavanones	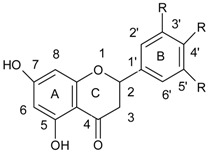
Anthocyanidins	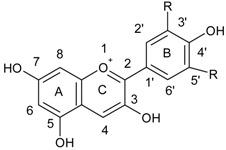
Isoflavonoids	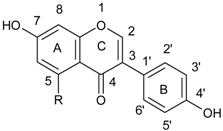
**Phenolic acids**	
Benzoic acid derivatives	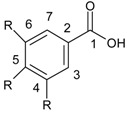
Cinnamic acid derivatives	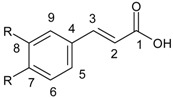

**Table 2:** Antistaphylococcal properties of flavonols, flavanols and phenolic acids.

Phenolic Compound	Proposed Mechanism of Action	Examined Strains	Synergism with Antibiotics	References
**Flavolons**				
Galangin	a	*S. aureus* NCTC 6571	Penicillin G	[38,41]
Morin	b	*S. aureus* clinical strains	-	[42]
Quercetin	c	MRSA clinical strains	Rifampicin Ciprofloxacin	[43,44,45]
Kaempferol	c	MRSA clinical strains	Rifampicin Ciprofloxacin Fluoroquinolone	[43,44,45,46]
**Flavanols and Derivatives**				
(−)-Epigallocatechin gallate	b,d	MRSA and MSSA clinical and standard strains	Oxacillin Ampicillin/Sulbactam Penicillin Imipenem Panipenem Meropenem Tetracyclin Oxytetracycline	[47,48,49,50,51,52,53]
(+)-catechin acyl derivatives	a	MRSA clinical strains	-	[40]
Epicatechin gallate	a,e	MRSA clinical strains	β-lactams Ampicillin Ampicillin/Sulbactam Cefazolin Cefepime Imipenem/Cilastatin	[4,37,54]
3-*O*-decyl-(+)-catechin	a	MRSA and MSSA clinical strains	-	[55]
(+)-catechin	e	MRSA clinical strains	Ampicillin Ampicillin/Sulbactam Cefazolin Cefepime Imipenem/Cilastatin	[4]
Catechin hydrate	nk	MRSA and MSSA clinical and standard strains	Clindamycin Erythromycin	[18]
**Phenolic Acids and Derivatives**				
Ferulic acid	a	*S. aureus* ATCC 6538	-	[56]
Coumaric acid	a	*S. aureus* ATCC 6538	-	[56]
Chlorogenic acid	a	*S. aureus* ATCC 6538	-	[56]
Protocatechuic acid ethyl ester	nk	MRSA and MSSA clinical and standard strains	Clindamycin	[5]
Caffeic acid	a	MRSA and MSSA clinical and standard strains	Clindamycin Erythromycin Cefoxitin	[16,57,58,59]

a—interaction with a cytoplasmic membrane, b—influence on the staphylococcal virulence factors, c—inhibition of bacterial topoisomerases activity, d—direct action on the bacterial cell wall, e—inhibition of bacterial gene expression, nk—not known.
